# Anomaly detection in gene expression via stochastic models of gene regulatory networks

**DOI:** 10.1186/1471-2164-10-S3-S26

**Published:** 2009-12-03

**Authors:** Haseong Kim, Erol Gelenbe

**Affiliations:** 1 Intelligent Systems Networks Group, Electrical and Electronic Engineering Department, Imperial College London, UK

## Abstract

**Background:**

The steady-state behaviour of gene regulatory networks (GRNs) can provide crucial evidence for detecting disease-causing genes. However, monitoring the dynamics of GRNs is particularly difficult because biological data only reflects a snapshot of the dynamical behaviour of the living organism. Also most GRN data and methods are used to provide limited structural inferences.

**Results:**

In this study, the theory of stochastic GRNs, derived from G-Networks, is applied to GRNs in order to monitor their steady-state behaviours. This approach is applied to a simulation dataset which is generated by using the stochastic gene expression model, and observe that the G-Network properly detects the abnormally expressed genes in the simulation study. In the analysis of real data concerning the cell cycle microarray of budding yeast, our approach finds that the steady-state probability of CLB2 is lower than that of other agents, while most of the genes have similar steady-state probabilities. These results lead to the conclusion that the key regulatory genes of the cell cycle can be expressed in the absence of CLB type cyclines, which was also the conclusion of the original microarray experiment study.

**Conclusion:**

G-networks provide an efficient way to monitor steady-state of GRNs. Our method produces more reliable results then the conventional *t*-test in detecting differentially expressed genes. Also G-networks are successfully applied to the yeast GRNs. This study will be the base of further GRN dynamics studies cooperated with conventional GRN inference algorithms.

## Background

Identifying the key features and dynamics of gene regulatory networks (GRNs) is an important step towards understanding behaviours of biological systems. Thanks to the development of high-throughput technology, the amount of microarray gene expression data has greatly increased, and numerous mathematical models attempt to explain gene regulations using gene networks [1,2]. Once a network structure is inferred, its dynamics needs to be considered. However, most methods focus on the inference of network structure which only provides a snapshot of a given dataset. Probabilistic Boolean Networks (PBNs) represent the dynamics of GRNs [3], but PBNs are limited by the computational complexity of the related algorithms [4].

In [5], a new approach to the steady-state analysis of GRNs based on G-Network theory [6,7] is proposed, while G-Networks were firstly applied to GRNs with simplifying assumptions concerning gene expression in [8]. However, the G-Network approach also exhibits specific difficulties because of the large number of parameters that are needed to compute their steady-state solution. Thus, in this study we reduce the number of model parameters on the basis of biological assumptions and focus on estimating two parameters in particular: the total input rate and steady-state probability of a gene.

A G-Network is a probabilistic queuing network having special customers which include positive and negative "customers", signals and triggers [6,7]. It was originally developed also as a model of stochastic neuronal networks [9] with "negative and positive signals or spikes" which represent inhibition and excitation. In terms of GRNs, a queue is a "place" in which mRNAs are stored, and an mRNA can be considered to be a "customer" of the G-Network. The positive and negative signals are interpreted as the protein activities such as transcription factors, inducers and repressors. Note that the customers or signals of the G-Network can be any biological molecules. However, in our study, we focus on behaviours of mRNAs because the available GRN data are usually mRNA expressions. Each queue has an input and service rates which represent a transcription and degradation processes, respectively. Our interest is to estimate the steady-state probability that a queue is busy, which corresponds to the probability that an mRNA is present, and we are also interested in the total mRNA input rate of each queue. To evaluation the accuracy of the proposed method, we generated a simple simulation dataset by using the stochastic gene expression models processed with the widely accepted Gillespie algorithm [10,11]. We also examine a real biological dataset obtained from the cell cycle of the budding yeast [12].

Although queueing theory is a common computational tool, G-Networks are an essential departure from queueing theory; in particular conventional queues could not be possibly applied to GRNs because the notion of inhibition does not exist in queueing theory but was introduced by G-Network theory. There are two other essential novelties in our work. First, our approach enables us to obtain the steady-state of GRNs with only polynomial computational complexity due to the product form solution of G-Networks; the computational cost due to large memory space and non-polynomial computational complexity are basic limitations in conventional methods such as PBN. Also our method can provide more reliable measures to detect differentially expressed genes in microarray analysis (as shown in our simulation study).

### G-networks and gene regulatory networks

The GRN model used in this study is the probabilistic gene regulatory model introduced in [5]. In this model, let *K*_*i*_(*t*) be integer-valued random variables which represent a quantity (mRNA) of the gene *i *at time *t*. If the *K*_*i*_(*t*) is zero, the gene *i *cannot interact with other genes. Then we have the following Probabilities,

where Λ_*i *_is the total input rate (sum of transcription rate, *λ*_*i *_and increment rate of mRNAs come from outside of system, *I*_*i*_), *μ*_*i *_is the service rate (e.g. Degradation rate of mRNAs). *o*(Δ*t*) → 0 as *t *→ 0. Let *r*_*i *_is representing the activity (signal process) rate of each gene *i*. Then 1/*r*_*i *_is the average time between successive interactions of gene *i *with other genes. If the *i*th gene interacts with other genes, the following events occur:

• With probability *P*^+ ^(*i*, *j*), gene *i *activates gene *j*; when this happens, *K*_*i*_(*t*) is depleted by 1 and *K*_*j*_(*t*) is increased by 1

• With probability *P*^- ^(*i*, *j*), gene *i *inhibits gene *j*; when this happens, both *K*_*i*_(*t*) and *K*_*j*_(*t*) are depleted by 1

• With probability *Q*(*i*, *j*, *l*) gene *i *joins with gene *j *to act upon gene *l *in excitatory mode, as a result of which both *K*_*i*_(*t*) and *K*_*j*_(*t*) are reduced by 1, while *K*_*l*_(*t*) is increased by 1

• With probability *d*_*i*_, which is defined as follow,

the signal of gene *i *exits the system so *K*_*i*_(*t*) is depleted by 1

Let's define a random process **K**(*t*) = [*K*_1_(*t*), ..., *K*_*n*_(*t*)], *t *≥ 0 and an *n*-vector of non-negative integers **k **= [*k*_1_, ..., *k*_*n*_]. The *P *(**k**, *t*) is the probability that **K**(*t*) takes **k **at time *t*, *P *(**k**, *t*) = *P *(**K**(*t*) = **k**). Then the probability that **K**(*t*) have **k **at time *t *+ Δ*t *is defined by

where  is a vector that the value of *i*th element is *k*_*i *_+ 1 (*k*_*i *_- 1) and *I*(*x*) is indicator function which is 1 if the condition, *x*, is satisfied or 0 other wise. The first and second terms describe the increment and decrement of the length of queue *i*, respectively. Third term is the probability that the gene *i *is activated but nothing is happened except queue *i *lose one mRNA. From fourth to sixth terms are the probabilities that gene *i *is activated and interacts with gene *j*. The rest terms of (1) represent the probabilities that the interaction of gene *i *and gene *j *affect the gene *l *(length of *l*th queue). Divide (1) by Δ*t *and introduce the equilibrium probability distribution of the system *P*(*k*) = *lim*_*t *→ ∞ _*P *(**k**, *t*) then we obtain following dynamic behaviour,

Now, let's consider following equations,  and 

Where *q*_*i *_(= /(*r*_*i *_+ )) represents the probability that gene *i *is expressed in steady-state. Using (2) and (3), E. Gelenbe showed the following product form is satisfied [5,7].

where for any subset *I *⊂ 1, ..., *n *such that *q*_*m*_*<*1 for each *m *∈ *I*, and *I*{*m*_1_, ..., *m*_|*I*|_}.

## Results and discussion

### Simple gene regulatory networks using stochastic gene expression model

**Figure 1:**
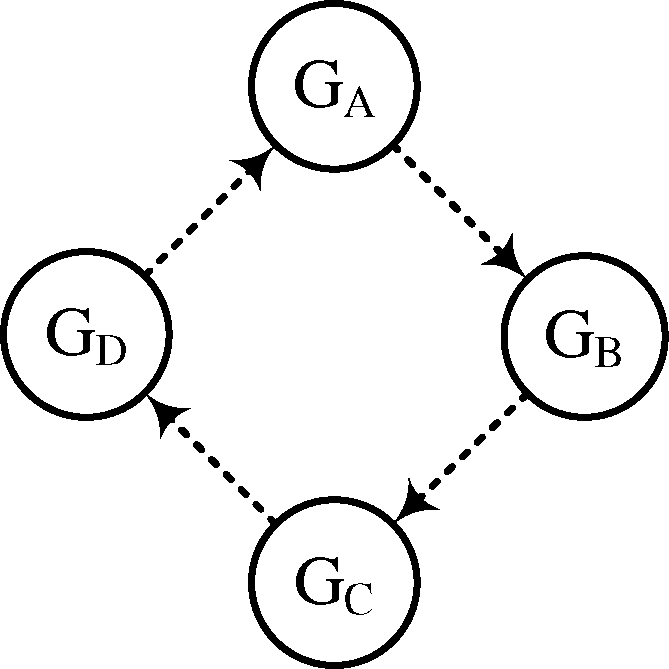
**Simple gene regulatory network structure**. The simulation study performed with the four gene GRN structure. Each gene inhibits its neighbor gene.

**Figure 2:**
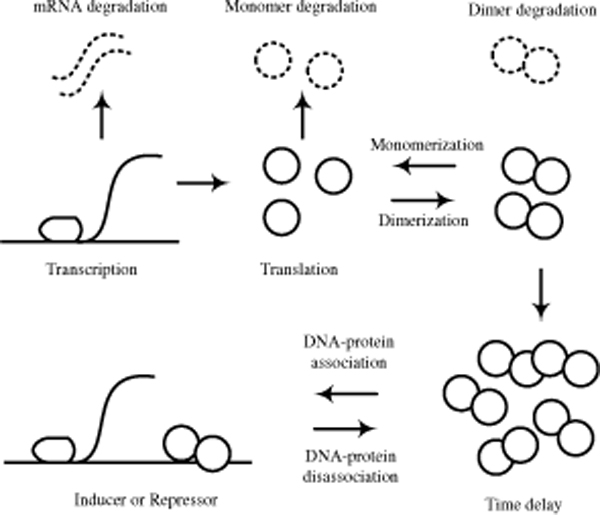
**Assumptions for the stochastic gene expressions**. There are total 10 processes (Transcription, Translation, mRNA degradation, Dimerization, Monomerization, Monomer degradation, Dimer degradation, Time delay for protein activation, DNA-protein association/disassociation) for the stochastic gene expression modeling.

In order to assess our G-Network model, we construct a simple GRN structure and generate the expression data using a synthetic stochastic gene expression model [13,14]. This stochastic gene expression model has several important features such as protein dimerization [15] and time delay for protein signalling [13]. Figure 1 shows the simulated network structure which is based on the following basic principles: the number of proteins per cell chases the number of mRNAs which in turn chases the number of active genes [14]. Figure 2 depicts the assumptions of our model and (5)~(11) give the corresponding processes (RPo: RNA open complex, Pro: promoter, R: mRNA, P: protein monomer, PP: protein dimmer, 0: degradation, *t*: time, and Δ*t*: time increment):

where *i*, *j *∈ {*A*, *B*, *C*, *D*} in Figure 1. In addition, we assume that proteins such as transcription factors and repressors require accumulation times for their activation [11,13], and use the modified Gillespie algorithm to generate the expression data [10,11]. The cell growth rate and cell volume are fixed, and we consider five cells. Detailed parameters are summarized in Table 1 with their references.

**Table 1:** Parameters of stochastic gene expression model

Parameters		Values	References
Transcription initiation	*λ*_2_	0.0025 *sec*^-1^	[16,22]
Translation initiation	*λ*_3_	0.0612 *sec*^-1^	[14,16]
mRNA degradation	*γ*_2_	0.00578 *sec*^-1^	[16]
Monomer degradation	*γ*_3_	0.00077 *sec*^-1^	[16,17]
Dimer degradation	*γ*_4_	0.00057 *sec*^-1^	[16,17]
Dimer association	*k*_*a*1_	0.1	[17]
Dimer dissociation	*k*_*d*1_	0.01	[17]
DNA-protein association	*k*_*a*2_	0.189	[18]
DNA-protein dissociation	*k*_*d*2_	0.0157	[18]
Burst size	*b*	10	[14,16]
Accumulation time of proteins	Δ*t*	0.1	[11]

Cell growth rate and the cell volume are fixed.

The transcription process in (5) follows an exponential distribution with transcription initiation rate *λ*_2 _[16]. The translation processes are given in (6) and include direct competition between the ribosome binding site and the RNAse-E binding site which degrade the mRNAs. Thus the translation process follows a geometric distribution with probability *p *and busting size *b *= *p*(1 - *p*) [13,16]. *T*_*D *_is the average time interval between successive competitions, and the number of surviving mRNAs *n*_2 _in the population after transcription is blocked with *n*_2 _= *n*_2,0 _. This is equal to *T*_*half *_= -(*log*(2)/*log*(*p*))*T*_*D *_[13]. Thus the translation initiation rate, *λ*_3 _= 1/*T*_*D*_, can be computed. The protein dimer association and disassociation rates are *k*_*a*2 _and *k*_*d*2_, respectively, as shown in (7) and (8) [17]. We also consider the DNA-protein association and disassociation rates (*k*_*a*1 _and *k*_*d*2 _in (9) and (10), respectively) [18]. The degradation rate of mRNA and of proteins are obtained by using the half-life of each molecule (11) [16,17].

We generate three sets of expression data (Dataset 1, 2, and 3); each dataset has two groups, the normal and the case group. These groups are obtained with the same parameter values except for the transcription initiation rate of *G*_*A *_in case group is 0.0012 *sec*^-1 ^which is half of the transcription rate in normal group, 0.0025 *sec*^-1^. Both groups are simulated during 3000 seconds. In order to compare these two groups, we perform not only the G-Network analysis but also the *t*-test which is widely used to find differentially expressed genes in microarray analysis. Datasets 1 and 2 consist of 50 samples each which are drawn from all the data points. In Dataset 1, the expression of *G*_*A *_is significantly different (*p*-value of *t*-test *<*0.01 in Table 2) while the difference of the *G*_*A *_expression in Dataset 2 is not significant. The third dataset consists of 500 samples which are randomly chosen from the original observations.

**Table 2:** Steady-state probability and total income rate of dataset showing significant *p*-value of *G*_*A*_

		Normal		Case				*t*-test
		*q*	Λ	*q*	Λ	*q*_*C*_/*q*_*N*_	Λ_*C*_/Λ_*N*_	*p*-value
Dataset 1 50 Samples	*G*_*A*_	0.512	0.765	**0.296**	**0.465**	**0.57**	**0.60**	0.000
	*G*_*B*_	0.517	0.785	0.595	0.775	1.15	0.98	0.123
	*G*_*C*_	0.502	0.765	0.546	0.875	1.08	1.14	0.311
	*G*_*D*_	0.487	0.735	0.563	0.875	1.15	1.19	0.127

Dataset 2 50 Samples	*G*_*A*_	0.445	0.675	**0.369**	**0.565**	**0.82**	**0.83**	0.202
	*G*_*B*_	0.423	0.615	0.556	0.765	1.31	1.24	0.016
	*G*_*C*_	0.472	0.675	0.432	0.675	0.91	1.00	0.439
	*G*_*D*_	0.510	0.755	0.525	0.755	1.02	1.00	0.661

Dataset 3 500 Samples	*G*_*A*_	0.474	0.725	**0.319**	**0.495**	**0.67**	**0.68**	0.000
	*G*_*B*_	0.503	0.745	0.584	0.775	1.16	1.04	0.000
	*G*_*C*_	0.460	0.695	0.443	0.705	0.96	1.01	0.304
	*G*_*D*_	0.521	0.765	0.541	0.785	1.03	1.02	0.122

Table 2 summarizes the results of the three datasets. In the case groups of Datasets 1 and 2, both the *q*_*A *_and Λ_*A *_have the lowest values among the four nodes while the *t*-test of the *G*_*A *_expression in Dataset 2 shows that it is not significant (*p*-value = 0.202). In the small sample results (Datasets 1 and 2), our method provides consistent results with large sample analysis (Dataset 3). The ratios (case/normal) also show that the *q*_*A *_and Λ_*A*_, in the case group, are smaller than one while the other ratios stay around one. In Dataset 3, the *p*-value of *G*_*B *_is significant along with that of *G*_*A *_because the expression of *G*_*A *_directly affects the expression of *G*_*B*_. However, *G*_*B *_is not the causal gene in this study. Our G-Network analysis reveals that only *G*_*A *_has lower *q *and Λ values than other nodes including *G*_*B*_. All these results concur with the simulation data generated with one half of the normal transcription rate.

### Modeling cell cycle gene regulatory networks in budding yeast

**Figure 3:**
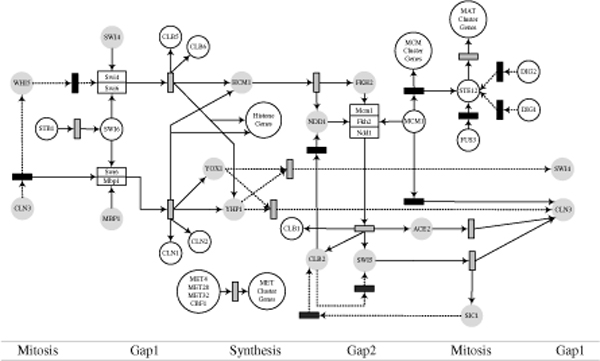
**Cell cycle regulatory network structure in budding yeast**. The genes are represented by circles. Complex molecules consisted of two more proteins are represented by a white rectangle. The gray and black boxes are transcription and post-transcription processes, respectively. Activation processes are depicted by the solid lines and inhibitions or repressions are shown by the dashed lines. The genes with gray circles are used to model the G-Networks.

**Figure 4:**
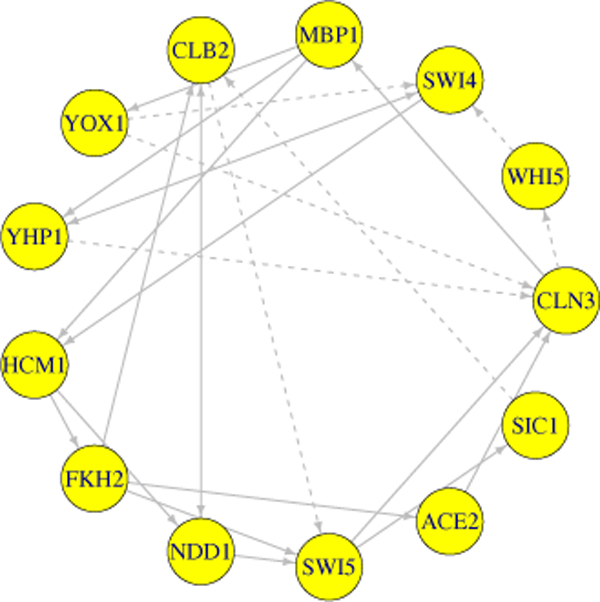
**Cell cycle regulatory network structure with selected 13 genes**. Each node represents a queue. Signals are transferred through the edges. Solid and dashed lines are positive and negative interactions, respectively.

The cell cycle regulated transcription and its overall controls have been studied in detail for budding yeast [19]. Recent developments in high-throughput microarray techniques help to reveal many of yeast genes controlling the cell cycle [20] which consists of four distinct phases: Gap1 (G1), Synthesis (S), Gap2 (G2), and Mitosis (M). The cells grow during their G1 and G2 phases and their DNA is replicated during the S phase. In the M phase, cell growth stops and the cell divides into two daughter cells that include nuclear division. Many genes are involved with specific cell cycle phases, but the number of key regulators that are responsible for the control of the cell cycle process is much smaller. Thus, based on published information, we build a cell cycle GRN with the key regulators in budding yeast as shown in Figure 3, although the relationships that contribute to the true regulatory network structure of the cell cycle still remain uncertain. Therefore we simplify the cell cycle network structure by selecting thirteen key regulatory genes (the gray circles in Figure 3) and connect the genes without regard to the transcriptional and post-transcriptional processes. Figure 4 shows the reconstructed regulatory network structure.

The activity of cyclin-dependent kinases (CDKs) plays an important role in controlling periodic events during cell cycle. Some studies of cell cycle with high-throughput technologies have suggested alternative regulation models of periodic transcription [20]. D. Olando et., al. [12] measured the transcription levels of cell cycle related genes with the use of Yeast 2.0 oligonucleotide array and determined the manner in which transcription factor networks contribute to CDKs and to global regulation of the cell-cycle transcription process. This microarray dataset is used in our study with the cell cycle network structure of Figure 4; it consists of two groups: one group is obtained from wild-type (WT) cells and the other is from cyclin-mutant (CM) cells which are disrupted for all S-phase and mitotic cyclins (mutate clb1, 2, 3, 4, 5, and 6).

**Figure 5:**
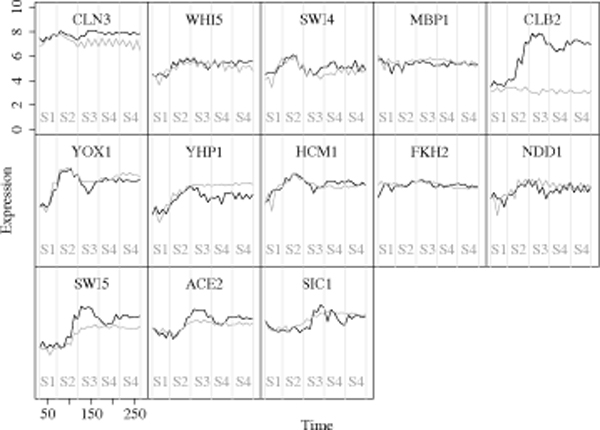
**Expression profiles of selected 13 genes**. The black and gray lines represent the wild-type (WT) and clb-mutant (CM) groups' expression levels.

The microarray data consist of a total of 30 data points taken over 270 minutes. We subdivide it into five states (groups), each consisting of 6 data points. The expression levels are transformed by taking the natural logarithm. Figure 5 depicts the transformed expression profiles of the 13 genes with 5 states. The black and gray solid lines are the expression profiles from WT and CM cells, respectively, and S1, S2, ..., S5 represent the five states. It is obvious that the profiles of CLB2 are different between WT and CM cells because the CM dataset is designed to monitor the cell cycle processes without the clb cyclines.

Table 3 summarizes the steady-state probabilities of 13 genes in the cell cycle GRN. All genes have similar steady-state probabilities in the WT and CM cell groups except for CLB2 in the CM group, which has a lower steady-state probability than the elements of the WT group: as shown in Table 3, the ratio of CM/WT is smaller than one (bold letter). This smaller probability can be explained by considering the experimental design of the CM dataset which is obtained without clb cyclines. Also, the original study of this dataset suggested alternative cell cycle regulatory pathways in [12] which had revealed that over 70% of the cell cycle related genes were expressed periodically without the clb cyclines. In our results, the steady-state probabilities of the CM group are consistent with that of the WT group. These results draw the same conclusion as the original study, i.e. that the steady-state of the 12 genes does not entirely depend on the expression of CLB2. Table 4 shows the estimated total input rate of the 13 genes. These results also show that only the input rates of CLB2 decrease in the CM group.

**Table 3:** Steady-state probability of the 13 genes in cell cycle GRNs

State	Cells	CLN3	WHI5	SWI4	MBP1	CLB2	YOX1	YHP1	HCM1	FKH2	NDD1	SWI5	ACE2	SIC1
S1	WT	0.880	0.813	0.829	0.839	0.784	0.99	0.803	0.843	0.855	0.836	0.799	0.99	0.99
	CM	0.878	0.814	0.818	0.848	0.770	0.99	0.802	0.842	0.864	0.839	0.787	0.99	0.99
	C/W	0.998	1.001	0.987	1.011	**0.981**	1.00	0.999	0.999	1.011	1.004	0.986	1.00	1.00

S2	WT	0.882	0.845	0.845	0.840	0.847	0.99	0.850	0.870	0.863	0.863	0.825	0.99	0.99
	CM	0.876	0.837	0.846	0.847	0.769	0.99	0.853	0.873	0.865	0.861	0.807	0.99	0.99
	C/W	0.994	0.990	1.000	1.008	**0.909**	1.00	1.004	1.004	1.002	0.998	0.978	1.00	1.00

S3	WT	0.890	0.840	0.826	0.846	0.886	0.99	0.844	0.855	0.863	0.854	0.871	0.99	0.99
	CM	0.880	0.846	0.820	0.849	0.751	0.99	0.863	0.863	0.869	0.870	0.840	0.99	0.99
	C/W	0.989	1.008	0.993	1.003	**0.847**	1.00	1.022	1.010	1.007	1.019	0.964	1.00	1.00

S4	WT	0.890	0.841	0.837	0.845	0.866	0.99	0.839	0.870	0.862	0.853	0.857	0.99	0.99
	CM	0.879	0.835	0.821	0.849	0.757	0.99	0.864	0.864	0.859	0.863	0.845	0.99	0.99
	C/W	0.988	0.993	0.982	1.005	**0.874**	1.00	1.029	0.994	0.996	1.012	0.986	1.00	1.00

S5	WT	0.891	0.850	0.837	0.846	0.877	0.99	0.839	0.869	0.862	0.856	0.865	0.99	0.99
	CM	0.869	0.830	0.823	0.842	0.756	0.99	0.862	0.862	0.857	0.861	0.845	0.99	0.99
	C/W	0.976	0.977	0.983	0.995	**0.862**	1.00	1.027	0.991	0.994	1.006	0.976	1.00	1.00

**Table 4:** Estimated total input rate of the 13 genes in cell cycle GRNs

State	Cells	CLN3	WHI5	SWI4	MBP1	CLB2	YOX1	YHP1	HCM1	FKH2	NDD1	SWI5	ACE2	SIC1
S1	WT	4.127	2.248	5.309	0.763	1.278	2.006	0.914	0.995	0.884	1.015	1.783	1.006	1.006
	CM	4.127	2.248	5.238	0.793	1.217	2.006	0.914	0.995	0.914	1.036	1.702	1.006	1.006
	C/W	1.000	1.000	0.987	1.040	**0.953**	1.000	1.000	1.000	1.034	1.020	0.955	1.000	1.000

S2	WT	4.187	2.339	5.521	0.763	1.430	2.006	0.995	1.036	0.854	0.995	1.945	1.006	1.006
	CM	4.187	2.309	5.521	0.793	1.187	2.006	0.995	1.036	0.854	1.056	1.743	1.006	1.006
	C/W	1.000	0.987	1.000	1.040	**0.830**	1.000	1.000	1.000	1.000	1.061	0.896	1.000	1.000

S3	WT	4.187	2.339	5.379	0.763	1.551	2.006	0.995	1.015	0.884	0.955	2.187	1.006	1.006
	CM	4.187	2.339	5.379	0.793	1.127	2.006	1.036	1.036	0.884	1.096	1.824	1.006	1.006
	C/W	1.000	1.000	1.000	1.040	**0.726**	1.000	1.041	1.020	1.000	1.148	0.834	1.000	1.000

S4	WT	4.187	2.339	5.450	0.763	1.490	2.006	0.975	1.036	0.854	0.955	2.106	1.006	1.006
	CM	4.187	2.309	5.379	0.793	1.157	2.006	1.036	1.036	0.854	1.076	1.864	1.006	1.006
	C/W	1.000	0.987	0.987	1.040	**0.776**	1.000	1.062	1.000	1.000	1.127	0.885	1.000	1.000

S5	WT	4.187	2.369	5.450	0.763	1.521	2.006	0.975	1.036	0.854	0.955	2.147	1.006	1.006
	CM	4.127	2.278	5.379	0.793	1.157	2.006	1.036	1.036	0.854	1.076	1.864	1.006	1.006
	C/W	0.986	0.962	0.987	1.040	**0.761**	1.000	1.062	1.000	1.000	1.127	0.868	1.000	1.000

## Conclusion

This paper has used the G-Network approach [5-8] to model GRNs. Two model parameters, the steady-state probability, *q*_*i*_, and the total input rate, Λ_*I*_, are estimated by determining the boundary of Λ_*i *_and using a grid search. We first use simulated gene expression data generated on the basis of a stochastic gene expression model. Two groups (normal and case) of expression data are examined. These two groups are exactly the same except for one parameter, the transcription initiation rate. We have observed that the G-Network based method is able to detect the abnormally expressed genes, while the *t*-test produces false positives. Then, using real data, we have observed that the steady-state probability of CLB2 is lower than that of other agents and concluded that the key genes of cell cycle regulation can be expressed without the clb cyclines; this result is consistent with the original experimental study.

However, the unchanged steady-state probabilities in all the five states may need to be considered, because the cell cycle has four phases (G1, S, G2, M) and expressions of genes involved with a specific phase are expected to be different from those in other phases. Also the small decrease rate and relatively large total input rates of CLB2 may require a more careful analysis of the G-Network approach in relation to cell cycle GRN structure. The manner in which we have used G-Network models in this paper did not currently include simultaneous interactions with three or more nodes. However this is not really a limiting effect of the model, since it suffices to include chain representations of dependencies in the G-Network model as has been done for neuronal networks [9] to cover excitatory and inhibitory effects that involve three or more nodes, and in fact random chains of nodes of any length. Although in this study the probabilities that gene *i *affect gene *j*, *P*^+ ^(*i*, *j*) and *P*^- ^(*i*, *j*) in (3), are fixed at the value one, we think that the conventional reverse engineering GRN methods using the "Ensemble" method [21] can provide these probabilities more accurately for an improved steady-state analysis of GRNs.

In conclusion, our study has illustrated the use of G-Networks as a new approach for the steady-state analysis of GRNs, and has shown their usefulness in obtaining quantities such as the effective transcription rate and the steady-state probabilities, using them to detect differentially expressed genes, thus introducing a new approach which differs from more conventional microarray analysis methods. Future research will investigate the ensemble approaches to GRNs [21] based on the G-Network methodology in [5], which will allow to infer GRN structures, and also to monitor their steady-state behaviour.

## Methods

Once a GRN structure is determined, it is necessary to estimate the total input rate (Λ_*i*_) of *i*th queue and its steady-state probability, (*q*_*i*_). For the simplicity, the probabilities, *P*^+ ^(*i*, *j*), *P*^- ^(*i*, *j*), and *Q*(*i*, *j*, *l*) in (3) are set to be one. Then, it can be rewritten as follows

In (12), the Λ_*i *_and *R*_*i *_is the total input (Λ_*i *_= *λ*_*i *_+ *I*_*i*_) and total output rates (*R*_*i *_= *r*_*i *_+ *μ*_*i*_), respectively.  is a function of activation probabilities of genes which affect to gene *i *positively and  is a function of activation probabilities of genes which affect to gene *i *negatively. We fix the *r*_*i *_as the number of out degrees of gene *i *and the degradation rate of mRNA, *i*, as a constant (Table 1) because the total output rate, *R*_*i *_is not our interest. Therefore, we need to estimate two parameters, the total input rate, Λ_*i*_, and the steady-state probability, *q*_*i*_.

Let  is the lower bound of the Λ_*i*_, which is larger than zero. The lower bound of total input is regarded as an initial transcription rate without any external input. In this study, we use  = 0.0025 [16]. The upper bound of Λ_*i *_ is obtained by assuming inputs from other nodes are zero and the queues fully work. That is

where the probabilities  and  are one.

Let  is the initial value of *q*_*i*_. Then  can be obtained as follow,

where *x*_*ij *_is the observed expression level (number of mRNAs) of *i*th gene at the *j*th observation and max(*x*_*ij*_) is the maximum value among all observed values of *i*th gene. Let Λ_*iu *_is a value of total input rate between the lower bound and the upper bound of Λ_*i *_(). Then the steady-state probability *q*_*i *_can be obtained numerically by solving (12) with the  and the Λ_*iu*_. Once the steady-state probability is determined, the log-likelihood of the given model can be computed by using (4) which is the same form of the likelihood of geometric distribution. It is known that the log-likelihood of geometric distribution is convex so we choose appropriate value Λ_*i *_which maximizes the log-likelihood function.

For each value of total input, Λ_*iu *_(), we compute the steady-state probability, *q*_*iu*_, with initial value, , and obtain the log-likelihood score, log *L*_*iu*_, which is used to choose the optimal *I *total input rate, ,

Note that the *q*_*iu *_is a numerical solution of (12) with initial value, , and total input rate, Λ_*iu*_. In order to compute , the initial value,  in (13) is substituted by  which is a numerical solution of (12) with initial value, , and total input rate, . Then the steady-state probability of gene *i*, *q*_*i*_, can be obtained by updating its value iteratively until the *d*^(*t*) ^<*δ *where *d*^(*t*) ^is the difference between  and  at *t*th iteration. In this study, *δ *is 0.0001.

## Competing interests

The authors declare that they have no competing interests.

## Authors' contributions

Haseong Kim developed the data analysis techniques including synthetic data generation and tested the models on the data. He wrote the first draft of the paper.

E. Gelenbe developed the G-Network models and the specific application of these models to GRNs. He rewrote the paper for submission, and then finalised the accepted paper in preparation for its publication.

## Note

Other papers from the meeting have been published as part of *BMC Bioinformatics* Volume 10 Supplement 15, 2009: Eighth International Conference on Bioinformatics (InCoB2009): Bioinformatics, available online at http://www.biomedcentral.com/1471-2105/10?issue=S15.
